# Data on optimum recycle aggregate content in production of new structural concrete

**DOI:** 10.1016/j.dib.2017.11.012

**Published:** 2017-11-04

**Authors:** Suvash Chandra Paul

**Affiliations:** Civil Engineering, Monash University Malaysia, Bandar Sunway, 47500 Subang Jaya, Selangor, Malaysia

**Keywords:** Recycle concrete, Mechanical strength, Creep, Shrinkage, Durability

## Abstract

This data presented herein are the research summary of “mechanical behavior and durability performance of concrete containing recycled concrete aggregate” (Paul, 2011) [1]. The results reported in this article relate to an important parameter of optimum content of recycle concrete aggregate (RCA) in production of new concrete for both structural and non-structural applications. For the purpose of the research various types of physical, mechanical and durability tests are performed for concrete made with different percentages of RCA. Therefore, this data set can be a great help of the readers to understand the mechanism of RCA in relates to the concrete properties.

**Specifications Table**

**Table:** 

Subject area	*Civil Engineering, Materials Science Engineering,*
More specific subject area	*Physical, Mechanical and Durability Properties*
Type of data	*Table, Images, Figures, Text File*
How data was acquired	*Laboratory Experiments of Physical, Mechanical and Durability Tests*
Data format	*Raw, Analyzed*
Experimental factors	*Four different percentages of volume fraction of recycle concrete aggregates are replaced with natural aggregate in production of new structural concrete.*
Experimental features	*Various volume of recycle concrete aggregates are blended with natural aggregate to investigate the physical, mechanical and durability properties*
Data source location	*Stellenbosch, South Africa*
Data accessibility	*The all data herein and relevant files are all available in this article*
Related research article	*SC, Paul. Mechanical behavior and durability performance of concrete containing recycled concrete aggregate. MSc Thesis, Stellenbosch University, Stellenbosch, South Africa, 2011.*

**Value of the data**

• The influence of replacement of recycle concrete aggregate in production of new structural concrete is discussed here
• This data set can also be used as a guideline for others to scrutinize the properties of recycle concrete aggregate
• The research data presented herein may be useful to manufacture different commercial elements using recycle concrete aggregate
• This data set may also encourage to recycle other wastes to minimize the dependency on natural resources

## 1 Data

This dataset reported herein were obtained from the experimental studies conducted on the different percentages of recycle concrete aggregate (RCA) replacement in the concrete mix and relate them to the influence of physical, mechanical and durability properties. The detailed of the dataset can also be found in [1], [2], [3]. Additionally, the available data presented by other researchers [4], [5], [6] were used to check the applicability of RCA in new concrete. A large number of cubes and cylinders (about 300) were prepared to examine the aforementioned properties of RCA. Finally, the properties of concrete made from RCA were compared with a reference concrete made with 100% natural aggregates (NA).

## 2 Experimental design, materials, and methods

The materials compositions for different RCA mixes used in the research are shown in Table 1.

**Table 1:** Materials compositions (% of total wt) of different RCA mixes.

Mix	Cement	NA	RCA	Sand	Water
RCA 0	0.12	0.41	0.00	0.39	0.07
RCA 15	0.12	0.35	0.06	0.40	0.07
RCA 30	0.12	0.21	0.20	0.40	0.07
RCA 50	0.12	0.29	0.12	0.40	0.07
RCA 100	0.12	0.00	0.41	0.40	0.07

The RCA was collected from the different sources of construction and demolition waste (C&DW) sites in the Western Cape region of South Africa. Concrete was then made by replacing different percentages (0%, 15%, 30%, 50% and 100%) of NA by RCA as they are assigned RCA0 to RCA100 in Table 1.

**Fig. 1:**
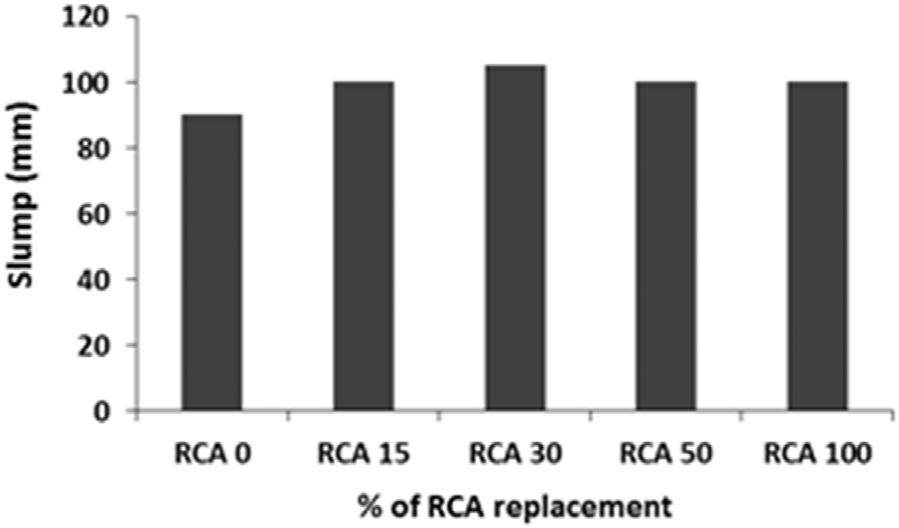
Measured slump value of concrete with different RCA replacement.

**Fig. 2:**
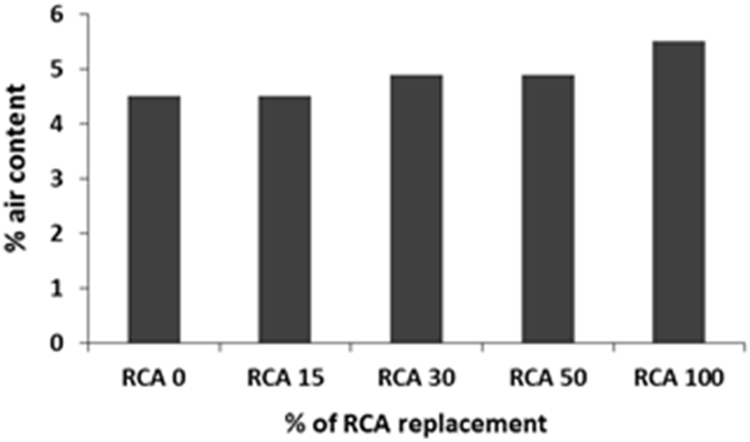
Percentages of air in fresh concrete mixes with different RCA replacement.

**Fig. 3:**
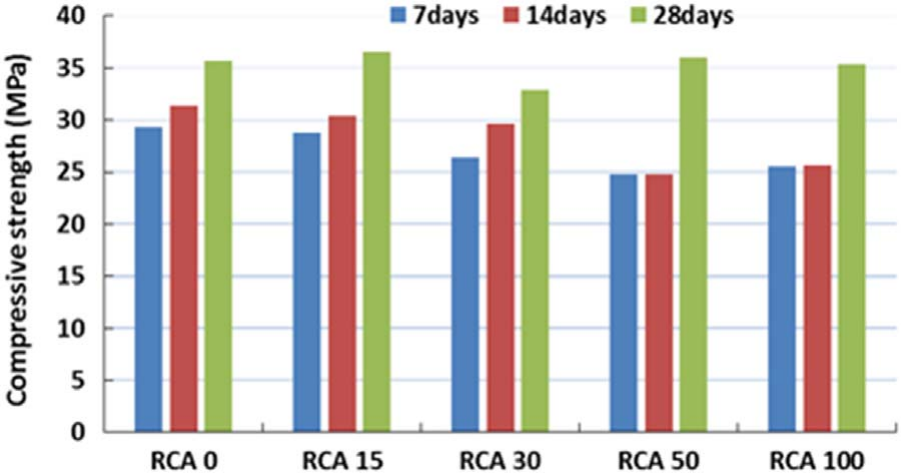
Compressive strength of concrete mixes with different RCA replacement.

**Fig. 4:**
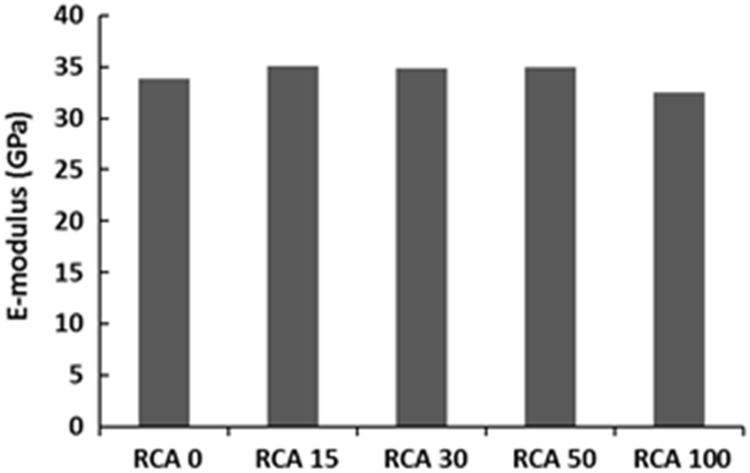
Young's modulus (E-modulus) of concrete mixes with different RCA replacement.

**Fig. 5:**
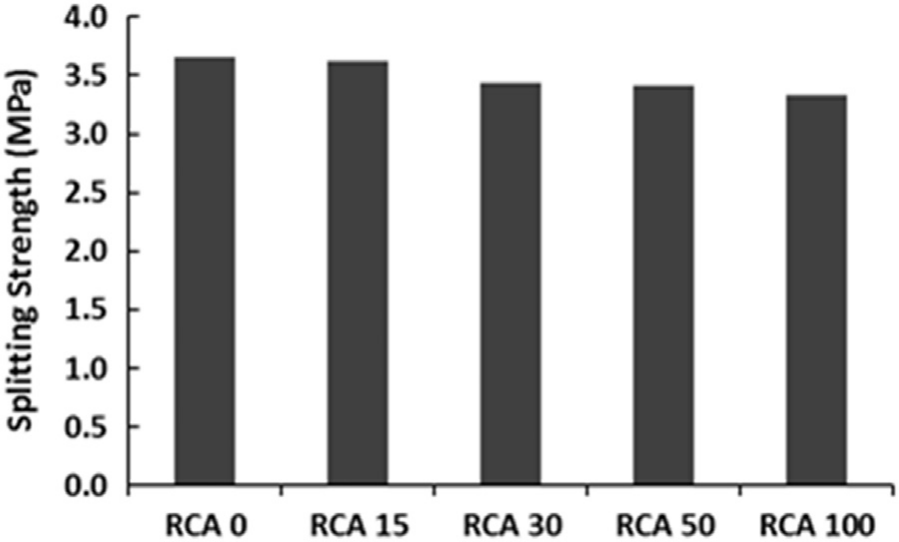
Splitting strength of concrete mixes with different RCA replacement.

**Fig. 6:**
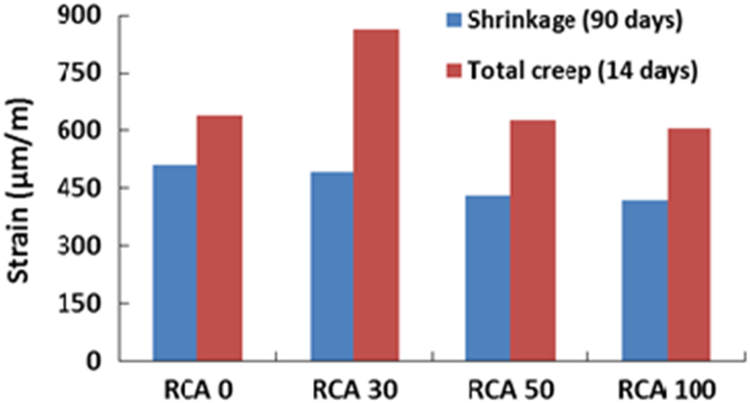
Dry shrinkage (at 90 days) and total creep strain (at 14 days) of different concrete mixes.

**Fig. 7:**
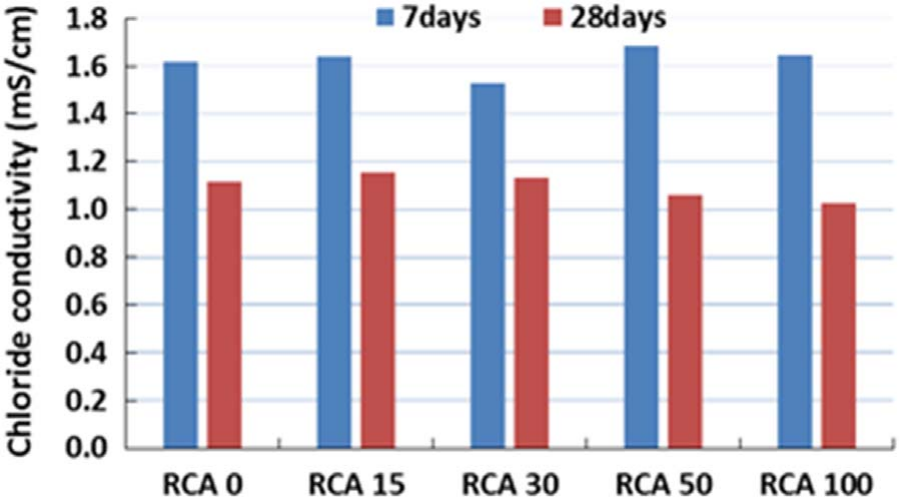
Chloride conductivity value of different concrete mixes.

**Fig. 8:**
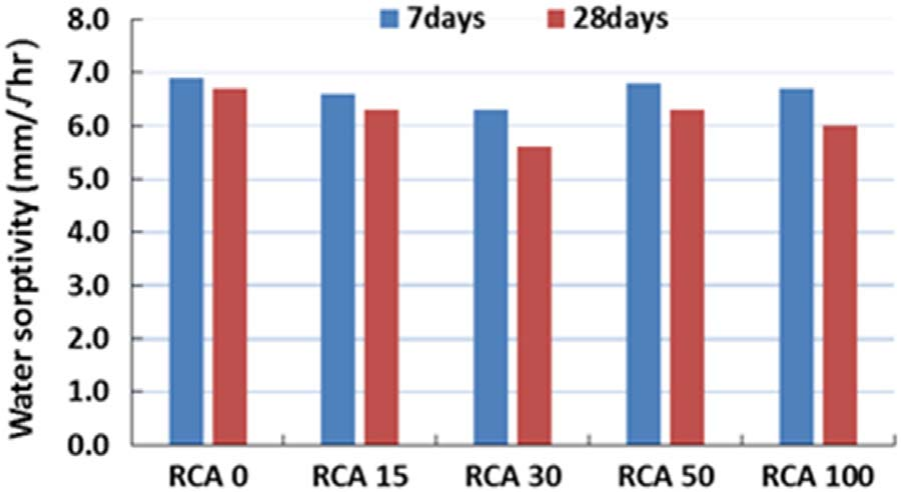
Water sorptivity value of different concrete mixes.

**Fig. 9:**
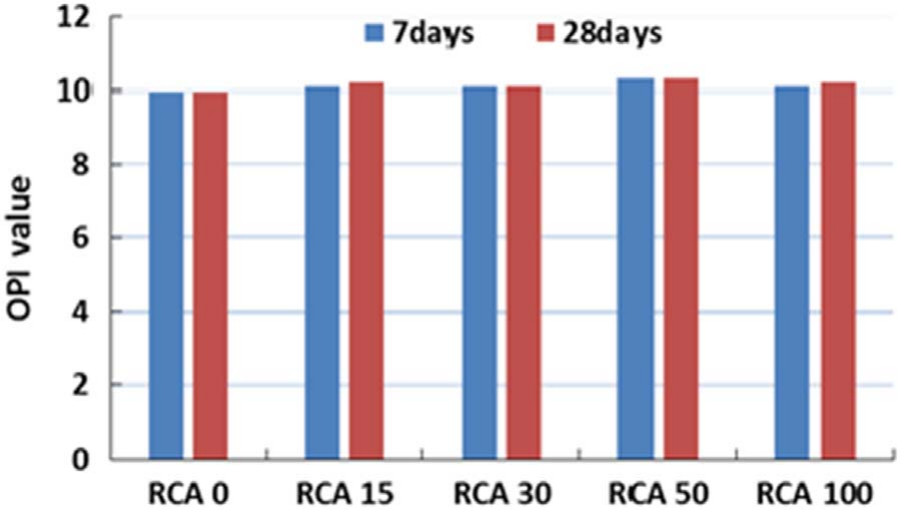
Oxygen permeability index (OPI) value of different concrete mixes.

The physical properties of aggregates such as relative density of aggregates, aggregate crushing value, flakiness index, and water absorption capacity were also measured and presented in Table 2. All mixing was performed under laboratory conditions. Slump test to check the workability of the concretes was performed as per SANS 5862:2006 [7], and air content as per SANS 6252:2006 [8]. After pouring concrete into the moulds, a vibration table was also used to ensure the compatibility of the fresh concrete. The compressive and splitting tensile strength of hardened concrete was determined on 100 mm and 150 mm cube specimens according to SANS 5863:2006 [9] and SANS 6253:2006 [10], respectively. Cylindrical specimens, 300 mm height × 150 mm diameter were used for evaluating the static E-modulus of RCA concrete in compression as per ASTM C469 [11]. The durability of concrete was investigated by means of chloride conductivity, water sorptivity and oxygen permeability index test according to the guideline provided in [12], [13]. Fig. 1, Fig. 2, Fig. 3, Fig. 4, Fig. 5, Fig. 6, Fig. 7, Fig. 8, Fig. 9 show the results when different percentages of RCAs were replaced with NA in production of new concrete. Furthermore, based on available data on RCA concrete the results were compared and details are discussed in [1], [2].

**Table 2:** Physical properties of NA and RCA.

Aggregate type	Relative density	Aggregates crushing value (%)	Flakiness index	Water absorption (%)
NA	2.72	11	25	0.60
RCA	2.63–2.77	10.8–12.5	19	3.2
