# Supplementary material for: Data on optimum recycle aggregate content in production of new structural concrete
Source: Data Brief. 2017 Nov 4;15:987–92. doi: 10.1016/j.dib.2017.11.012 (PMC5684096; doi:10.1016/j.dib.2017.11.012)
Supplement: Supplementary file 1 — Supplementary material [file mmc1.docx]

**Conflict of interest**

No potential conflict of interest was reported by the author.
